# Necroptosis does not drive disease pathogenesis in a mouse infective model of SARS-CoV-2 in vivo

**DOI:** 10.1038/s41419-024-06471-6

**Published:** 2024-01-30

**Authors:** Stefanie M. Bader, James P. Cooney, Reet Bhandari, Liana Mackiewicz, Merle Dayton, Dylan Sheerin, Smitha Rose Georgy, James M. Murphy, Kathryn C. Davidson, Cody C. Allison, Marc Pellegrini, Marcel Doerflinger

**Affiliations:** 1 The Walter and Eliza Hall Institute of Medical Research, Melbourne, VIC 3052 Australia; 2 Department of Medical Biology, University of Melbourne, Melbourne, VIC 3050 Australia; 3 Department of Anatomic Pathology, Faculty of Veterinary and Agricultural Sciences, University of Melbourne, Werribee, VIC 3030 Australia; 4 Drug Discovery Biology, Monash Institute of Pharmaceutical Sciences, Monash University, Parkville, VIC 3052 Australia

**Keywords:** Viral infection, Tumour-necrosis factors

## Abstract

Necroptosis, a type of lytic cell death executed by the pseudokinase Mixed Lineage Kinase Domain-Like (MLKL) has been implicated in the detrimental inflammation caused by SARS-CoV-2 infection. We minimally and extensively passaged a single clinical SARS-CoV-2 isolate to create models of mild and severe disease in mice allowing us to dissect the role of necroptosis in SARS-CoV-2 disease pathogenesis. We infected wild-type and MLKL-deficient mice and found no significant differences in viral loads or lung pathology. In our model of severe COVID-19, MLKL-deficiency did not alter the host response, ameliorate weight loss, diminish systemic pro-inflammatory cytokines levels, or prevent lethality in aged animals. Our in vivo models indicate that necroptosis is dispensable in the pathogenesis of mild and severe COVID-19.

## Introduction

SARS-CoV-2 infection predominantly causes mild disease, but a proportion of people develop a severe inflammatory response that leads to long term sequelae and death. Due to its high transmissibility, SARS-CoV-2 quickly became a pandemic agent [1]. The extent and magnitude of the pandemic compromises the efficacy of vaccines and antiviral agents in preventing all morbidity and mortality amongst diverse populations. This underscores the need for continuing research to identify novel intervention opportunities that can mitigate severe COVID-19.

Inflammation and cell death have been proposed to be central drivers of disease pathology in COVID-19 patients (as reviewed in [2, 3]), and SARS-CoV-2 infection was reported to induce the activation of key mediators of the host cell death machinery across a range of cell types (reviewed in [4]). Necroptosis, a lytic form of cell death, leads to the release of damage associated molecular patterns (DAMPs), causing immune activation, cytokine release and inflammation [5], including the secretion of IL-1β [6, 7], a cytokine that is associated with severe COVID-19 [8]. Necroptosis can be induced by death receptor signaling, including CD95 (FAS, Apo-1) [9], TNF receptor 1 (TNFR1), TNFR2 [10, 11], and TNF-related apoptosis-inducing ligand receptors 1/2 (TRAILR1 and TRAILR2) [12]. Toll-like receptor (TLR) activation can also lead to necroptosis through TIR domain-containing adapter-inducing interferon-β (TRIF) or MyD88 signaling [13]. Upon death receptor activation or toll-like receptor stimulation, the downstream adapter protein, Receptor Interacting Protein Kinase 1 (RIPK1) is engaged to differentially regulate three potential outcomes: (a) activation of NF-κB (b) induction of apoptosis and (c) induction of necroptosis. Necroptosis involves activation of RIPK1 and subsequent activation of RIPK3 within a heterooligomeric cytoplasmic assembly termed the necrosome, but only in the absence of caspase-8 function [12, 14]. Caspase-8 constrains the activity of RIPK3 and abrogates necroptosis. Certain viral infections can inhibit caspase-8 and therefore prime cells for necroptosis [15]. When circumstances conspire to activate RIPK3, this kinase phosphorylates the pseudokinase Mixed Lineage Kinase Domain-Like (MLKL) promoting its dissociation from RIPK3, oligomerization, plasma membrane translocation and permeabilization leading to cell death [16–20].

Recently, ‘priming’ of cells by type I and II interferon signaling was shown to induce MLKL protein expression [21, 22], suggesting potential relevance of necroptosis in the pathogenesis of inflammatory conditions, including viral infections associated with extensive interferon signaling [23]. Several reports have indicated that MLKL is phosphorylated across a range of cells during SARS-CoV-2 infection, including macrophages, lung epithelial cells, neutrophils, platelets, adipocytes, pancreatic islets and adrenal glands [24–28]. However, MLKL phosphorylation is necessary but not sufficient for the induction of necroptosis [17, 29]. Reports implicating necroptosis in COVID-19 rely on in vitro associations, post-mortem analyses and contrived transgenic mouse models where the human entry receptor for SARS-CoV-2, ACE2 (hACE) is aberrantly expressed under the control of various cell specific enhancer/promoters [30, 31]. These transgenic models do not recapitulate human disease, with the majority of mice dying of encephalitis [31]. Various models based on the delivery of hACE2 via a preceding adeno-associated virus (AAV) [32] or adenovirus infection (AVV) [33–35] have also been described. However, the introduction of a viral vector not only adds additional complexities but may also effectively trigger inflammatory responses. Presently, there is insufficient evidence to unequivocally implicate necroptosis in COVID-19 pathogenesis.

We address this controversy by using in vivo models of mild and severe COVID-19. We infected MLKL-deficient mice with SARS-CoV-2 and found no abrogation of disease compared to WT animals. This supports the notion that necroptosis is not a driver of severe COVID-19. Novel host-directed interventions are being avidly pursued for the management of severe COVID-19 and our study indicates that necroptosis is not a viable target.

## Results

### Loss of MLKL does not impact viral dissemination or immune response to a clinical isolate of SARS-CoV-2 in vivo

**Fig. 1:**
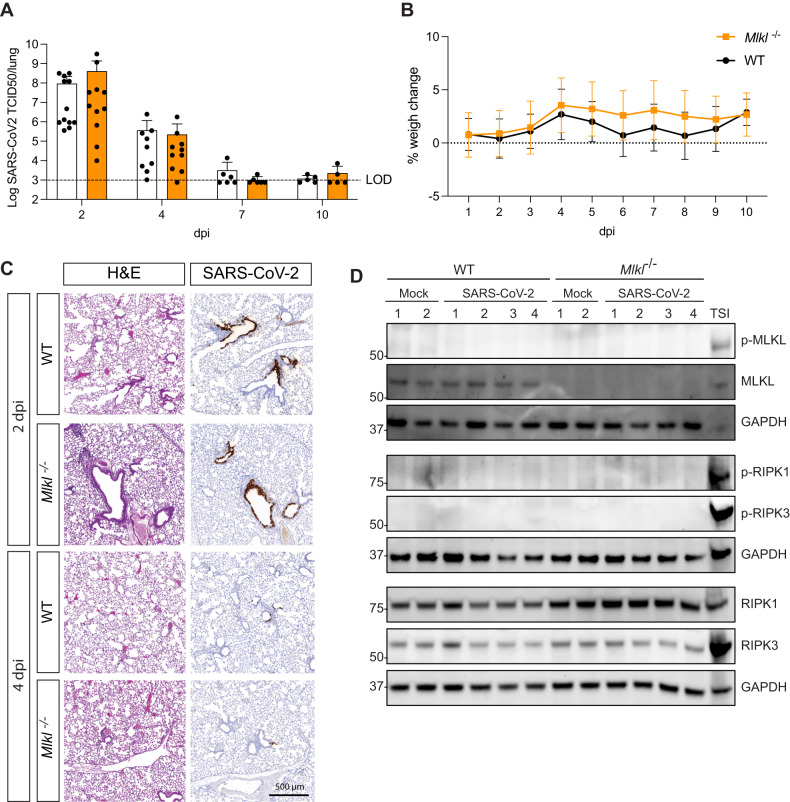
Necroptosis does not contribute to viral clearance or disease upon infection with a clinical isolate of SARS-CoV-2 (P2). **A**, **B** Mice were challenged intranasally with 10^4^ TCID50 of a clinical SARS-CoV-2 isolate (P2) **A** Animals were euthanised at defined days post-infection (dpi) and lungs were collected for viral quantification by TCID50 assay. (*n* = 5–12 mice per group, pooled from 2 independent experiments) **B** Daily percent weight change of infected animals compared to initial weight. (*n* = 4–6 mice per group) **C** Lungs were collected and fixed for histological analysis at 2 and 4 dpi. Representative images of hematoxylin and eosin (H&E) and immunohistochemistry (IHC) stained lungs with SARS-CoV-2 nucleocapsid. Histological images are representative of at least 3 animals. Scale bar = 500 µm. **D** Western blot analysis of homogenized lungs from wild-type (WT) and MLK knockout (*Mlkl*^−/−^) animals 3 days after P2 infection. Samples were probed for phosphorylated MLKL (p-MLKL), MLKL, Glyceraldehyde 3-phosphate dehydrogenase (GAPDH) as a house-keeping gene, phosphorylated RIPK1 (p-RIPK1), phosphorylated RIPK3 (p-RIPK3), RIPK1 and RIPK3. Whole cell lysates of bone marrow derived macrophages treated with Tumor Necrosis Factor (TNF), Smac mimetic IAP antagonist and pan-caspase inhibitor were used as a positive controls for necroptosis (*n* = 4 mice per group and are representative of 2 independent experiments).

To determine if necroptosis enables or potentially mitigates SARS-CoV-2 viral dissemination, we infected wild-type (WT) and MLKL-deficient mice (*Mlkl*^−/−^) with a clinical isolate of SARS-CoV-2 that was minimally passaged in mice (once, P2). P2 has been found through its N501Y mutation by genomic surveillance of infected Australians. Its N501Y has been correlated with viral adaptation to mouse ACE2 (mACE2) [36] and isolates carrying the substitution are able to infect and replicate in murine cells and have been successfully used in mouse models previously [37, 38]. We measured viral loads in the lungs on days 2, 4, 7 and 10 post infection (dpi) using a TCID50 assay (Fig. 1A). No differences were detected in viral clearance kinetics, as WT and *Mlkl*^−/−^ mice displayed similar viral titers with a peak at day 2 and almost complete clearance by day 10. Infection with SARS-CoV-2 did not lead to severe disease pathology, as mice did not display loss in body weight, a common surrogate marker for disease severity in mouse models [39] (Fig. 1B).

To interrogate possible differences in the cellular host response to SARS-CoV-2 infection between WT and *Mlkl*^−/−^ mice, we compared lung histology in animals infected at 2 and 4 dpi. Infection caused a similar response in WT and *Mlkl*^−/−^ mice, characterized by moderate bronchiolar epithelial necrosis and mild perivasculitis at 2 dpi, and mild vasculitis and fibrinoid changes in the vessels at 4 dpi. SARS-CoV-2 nucleocapsid staining shows that infection with P2 is localized only to the bronchiolar epithelium, with loads decreasing at 4 dpi compared to earlier time points (Fig. 1C). Using immunohistochemistry, we stained sections for myeloperoxidase (MPO), CD3 and F4/80 and did not observe any differences between infected WT and *Mlkl*^−/−^ animals (Fig. S1A).

To interogate necroptosis signaling during infection, we performed western blot analysis of mock and P2 infected lungs at 3 dpi. Consistent with low viral titers and limited bronchiolar epithelial spread in P2 infected mice, SARS-CoV-2 nucleocapsid staining was not detectable by western blotting across two large cohort of animals. Infection with the P2 SARS-CoV-2 clinical isolate was not associated with phosphorylation of MLKL (p-MLKL) in lung homogenates. The absence of p-MLKL aligned with a lack of phosphorylation of the upstream activators of necroptosis, RIPK1 and RIPK3 (Fig. 1D). Collectively, these results indicate that necroptotic signaling is not occurring during mild SARS-CoV-2 infection. We also did not observe any significant alterations in MLKL expression. This observation is noteworthy as MLKL expression is known to correlate with elevated levels of type I and II interferons [40–42] and P2 infection did not appear to induce a pronounced interferon response [43].

### Necroptosis does not affect inflammation or disease severity in animals infected with a more pathogenic, mouse passaged strain

**Fig. 2:**
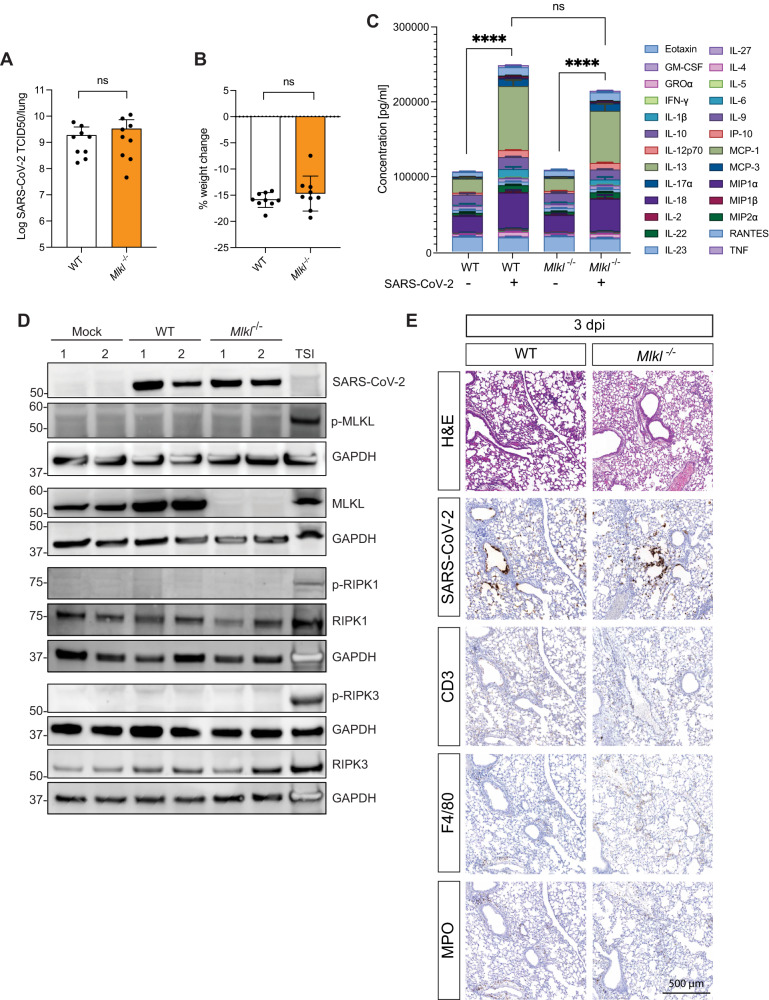
Severe disease caused by a mouse-adapted strain of SARS-CoV-2 (P21) is not affected by *Mlkl* knockout. **A**, **B**, **C** WT and *Mlkl*^−/−^ mice were challenged intranasally with 10^4^ TCID50 of SARS-CoV-2 P21 and monitored 3 days post infection for **A** lung viral burden using TCID50 assay **B** percent weight change compared to initial weight (n = 9 mice per group), and **C** the sum of twenty-six cytokines/chemokines expressed in supernatants of lung homogenates (*n* = 4 mice per group; mean ± SD of each cytokine are shown). **D** Western blot analysis of homogenized lungs from mock (intranasal DMEM inoculation), wild-type (WT) and MLK knockout (*Mlkl*^−/−^) animals 3 days after intranasal P21 infection (10,000 TCID50). Samples were probed for phosphorylated MLKL (p-MLKL), MLKL, Glyceraldehyde 3-phosphate dehydrogenase (GAPDH) as a house-keeping gene, phosphorylated RIPK1 (p-RIPK1), phosphorylated RIPK3 (p-RIPK3), RIPK1, RIPK3 and SARS-CoV-2 nucleocapsid. Whole cell lysates of bone marrow derived macrophages treated with Tumor Necrosis Factor (TNF), Smac mimetic IAP antagonist (LCL-161) and pan-caspase inhibitor (IDN-6556) were used as positive controls for necroptosis (*n* = 2 mice per group and are representative of 2 independent experiments). **E** Representative images of hematoxylin and eosin (H&E) and immunohistochemistry (IHC) stained lungs with SARS-CoV-2 nucleocapsid. Mice were challenged intranasally with 10^4^ TCID50 of SARS-CoV-2 P21 and lungs were collected and fixed for histological analysis at 3 dpi. Histological images are representative of at least 3 animals. Scale bars = 500 µm. Unpaired two-tailed student’s *t*-test (**B**) after performing log_10_ transformation (**A**) and one-way ANOVA with multiple comparisons (**C**).

We next sought to clarify a potential role for necroptosis during severe SARS-CoV-2 disease. We infected WT and gene-targeted animals with a derivative of the clinical isolate that was passaged in vivo 21 times (P21) [43]. This mouse-adapted, “P21” SARS-CoV-2 strain caused severe disease, characterized by increased release of pro-inflammatory cytokines, inflammation in the lungs and weigh loss over 2–5 dpi, with a peak at 3 dpi. This mouse adapted virus maintained a requirement for murine ACE2, as m*Ace2*^−/−^ mice were refractory to infection (Fig. S2A, B). P21 caused similar disease in both WT and *Mlkl*^−/−^ animals (Fig. S2C). At the peak of infection (3 dpi), WT and *Mlkl*^−/−^ mice had comparable viral loads and displayed the same level of weight loss (Fig. 2A, B). Quantification of the levels of twenty-six cytokines and chemokines in the lungs of WT and MLKL-deficient mice showed that mice lacking essential necroptotic signaling components responded similarly to WT animals upon infection (Fig. 2C). Importantly, this analysis included cytokines reported to be key mediators of human COVID-19 disease such as TNF, IFNγ and IL-1β [44–47] (Fig. S2D). Since type I and II interferons (IFN) have been linked to increasing MLKL protein expression [21, 22], we investigated whether P21 SARS-CoV-2 infection altered protein levels of MLKL and its activated, phosphorylated form (p-MLKL). Western blot analysis of lungs from WT animals showed increased levels of MLKL upon SARS-CoV-2 infection, but no p-MLKL was detected in either mock or infected animals **(**Fig. 2D**)**. Expression levels of the upstream activators of MLKL, RIPK1 and RIPK3, were similar between MLKL-deficient, WT and mock animals, and were also not upregulated upon SARS-CoV-2 infection. The absence of phosphorylation of RIPK1 or RIPK3 in the P21 infected mice provides additional evidence that SARS-CoV-2 does not induce necroptosis in the context of severe COVID-19.

Histological examination of lungs of animals infected with P21 at 3 dpi revealed comparable levels of perivasculitis, multifocal bronchial epithelial necrosis, and infiltration of inflammatory cells in both WT and *Mlkl*^*−/−*^ animals. During the initial stages of infection, P21 primarily invades and replicates within the bronchiolar epithelium (Fig. 2E). As disease progresses (>3dpi), the virus spreads further to alveolar pneumocytes (Fig. S3A). Quantification of inflammatory cells, alveolar septal thickening and interstitial pneumonia did not reveal differences in MLKL knockout mice compared to WT (Fig. S3B, C). Staining for immune cells showed comparable levels of myeloperoxidase (MPO), CD3 and F4/80 positive cells between WT and *Mlkl*^−/−^ mice at all examined time points (Figs. 2E and S3A).

### Exacerbated age-dependent disease is not triggered by necroptosis

**Fig. 3:**
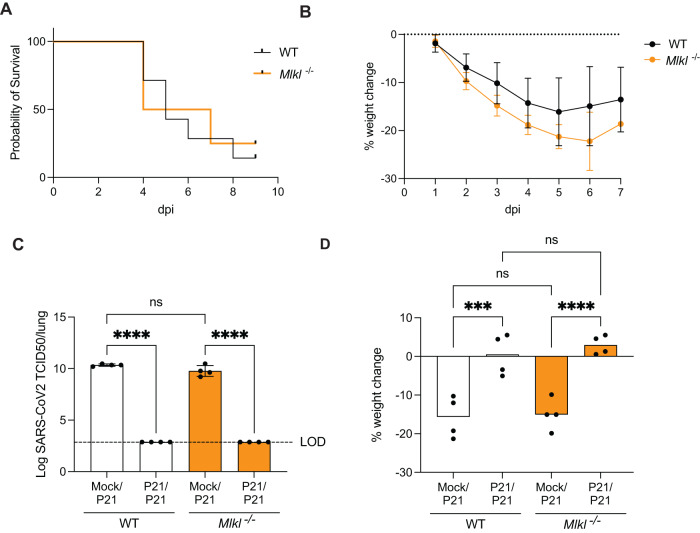
Lack of necroptosis pathways does not affect age-related disease severity or response to re-challenge. **A**, **B** Aged C57BL/6 and *Mlkl*^−/−^ mice (6–8 months) were intranasally inoculated with 10^4^ TCID50 SARS-CoV-2 P21 and monitored for **A** the proportion of mice that became moribund, reaching humane endpoint and **B** daily percentual weight change over time, relative to the initial weight. (*n* = 6–8 mice per group; Data is representative of 2 independent experiments) **C**, **B** Mock or P21-infected C57BL/6 and *Mlkl*^−/−^ mice were rechallenged 28 days later with P21 and analyzed 3 days post re-challenge for **C** lung viral load (TCID50) and **D** percent weight change, compared to weight before second infection (*n* = 4 mice per group; Data are representative of 2 independent experiments). One-way ANOVA with multiple comparisons (**D**), after log_10_ transformation (**C**) was performed. Mean ± SD are shown. ***p < 0.001, ****p < 0.0001.

In humans, age is a major risk factor for severe COVID-19 [48, 49], but the exact mechanisms leading to increased morbidity and mortality in the older population remain elusive. Necroptotic signaling has been associated with age related diseases (reviewed in [50, 51]), suggesting that necroptosis might contribute to severe COVID-19 in aged individuals. We therefore infected aged WT and *Mlkl*^−/−^ mice (>6-month-old) with the more pathogenic, mouse-adapted P21 strain. Mice succumbed to SARS-CoV-2 infection at 3–5 dpi, irrespective of their genotype, and weigh loss was indistinguishable between WT and *Mlkl*^−/−^ animals (Fig. 3A, B). Collectively, loss of MLKL did not alter disease outcome in young or aged mice infected with a highly pathogenic mouse-adapted SARS-CoV-2 strain.

### Necroptosis does not shape adaptive immunity to SARS-CoV-2

We next sought to clarify a potential role for necroptosis in the adaptive immune response to SARS-CoV-2 infection. A recent report suggested a role for MLKL in supporting the antibody response to lung infection with *Streptococcus pneumoniae* [52]. To examine this, we re-challenged mice with SARS-CoV-2 one month after initial infection with P21. Regardless of mouse genotype, SARS-CoV-2 was undetectable in re-challenged animals (Fig. 3C). Initial infection with P2 protected WT and MLKL-deficient animals equally against SARS-CoV-2 re-challenge (Fig. S3D), including challenge with P21 (Fig. 3D), indicating that adaptive immune responses to SARS-CoV-2 are sufficient to protect against re-infection even in the absence of MLKL.

## Discussion

Recent studies have proposed a role for necroptosis in the detrimental inflammatory and cytokine responses associated with severe COVID-19 [24, 25, 28, 33, 53, 54]. Our data challenges this view, and we provide genetic in vivo evidence that necroptosis does not contribute to SARS-CoV-2 viral dissemination, disease pathology and early innate or adaptive immune responses.

Using a model of mild disease, we show that a patient-derived, circulating clinical SARS-CoV-2 isolate equally infects WT and MLKL-deficient mice generating the same viral titers and clearance kinetics. Histology of infected lungs could not detect any pathological differences between the genotypes, indicating that necroptosis does not contribute to outcomes in mild disease. In humans, SARS-CoV-2 infection is known to cause a broad range of pathology which can ultimately lead to death. To study the effects of necroptosis in severe COVID-19, we infected WT and *Mlkl*^*−/−*^ mice with a mouse-adapted SARS-CoV-2 strain (termed P21), which was generated by passaging the P2 clinical isolate [43]. In WT animals, P21 infection caused an exacerbated inflammatory response characterized by high viral loads, weight loss, lung pathology and a significant increase in pro-inflammatory cytokines in the lungs, including IL-1β and IL-18. These cytokines are often linked to necroptotic cell death [5]. MLKL-deficient animals did not show differences in any of these disease parameters upon P21 infection compared to WT animals, indicating that necroptotic cell death is not involved in the exacerbated inflammation associated with severe SARS-CoV-2 disease. While our models allow precise genetic dissection of necroptosis and its involvement in the varying degrees of pathogenesis caused by SARS-CoV-2, it is conceivable that necroptosis may play a role in individuals with genetic aberrations or conditions that cause dysregulation or a gain of function in this pathway.

The lack of difference in the pro-inflammatory cytokines between WT and *Mlkl*^*−/−*^ is in line with previous in vivo studies [33]. Li et al. utilized an adenovirus model overexpressing hACE2 to infect *Mlkl*^*−/−*^*, Ripk3*^*−/−*^ *and Zbp1*^*−/−*^ knockout animals. The study found that while deletion of *Zbp1* or *Ripk3* reduced immune cell infiltration and the cytokine response upon SARS-CoV-2 infection, *Mlkl*^*−/−*^ mice were similar to WT controls. This, together with our results, indicates that while RIPK3 and ZPB1 can act upstream of MLKL activation and necroptosis in certain scenarios [55], during SARS-CoV-2 infection these proteins are likely exerting their necroptosis-independent roles, leading to pro-inflammatory cytokine release and inflammation [56, 57].

Although the immune response to SARS-CoV-2 infection and disease outcome in MLKL knockout animals did not differ from wild-type (WT), we found increased expression of MLKL in WT mouse lungs infected with SARS-CoV-2 P21. This is consistent with reports that show a role for type I and II IFN causing increased expression of MLKL protein levels in epithelial and other infected immune cells [40–42]. Infiltration of immune cells and concomitant increase of pro-inflammatory cytokines in the lungs of infected animals is likely to contribute to the increased protein levels of MLKL. This is in line with results of our previous work, where we observed an upregulation of both *Mlkl* and *Zbp1* upon infection with our mouse adapted strain (P21) in a bulk RNAseq analysis of lungs [43]. However, while MLKL expression is necessary for a cell to undergo necroptosis, the level of expression does not predict whether necroptosis occurs. Likewise, while phosphorylation of MLKL is a hallmark of necroptosis pathway activation, the presence of p-MLKL does not necessarily predict whether a cell is undergoing necroptosis, since a threshold amount of p-MLKL translocated to the plasma membrane is the critical requirement for necroptosis [17, 19, 29]. Furthermore, robust detection of p-MLKL in immunofluorescence and immunohistochemistry studies is known to be challenging owing to low abundance of p-MLKL, low sensitivity and specificity of available antibodies, the disruption of epitopes by common fixatives, and the omission of positive and negative (knockout, dephosphorylated) controls [58, 59]. Consequently, it is unsurprising that reports on the activation status of MLKL during SARS-CoV-2 infection have been contradictory. While Li and colleagues reported phosphorylated MLKL (p-MLKL) staining in the lungs of HFH4-hACE2 animals and in post-mortem tissues of COVID-19 patients [45], Xu et al. observed upregulation of MLKL in lungs of patients with severe COVID-19, but could not detect p-MLKL [24]. Using Calu-3 cells, Li et al. reported that MLKL activation leads to IL-1β release in infected cells, consistent with recent reports showing that plasma membrane disruption by MLKL can lead to IL-1β release through efflux of potassium ions and subsequent NLRP3 activation [6, 7]. Our results suggest that, during SARS-CoV-2 infection in vivo, the contribution of necroptosis to IL-1β secretion is either minor or redundant, since infected WT and *Mlkl*^−/−^ mice show similar IL-1β levels in the lungs.

We have shown previously that infection with *Mycobacterium tuberculosis* (*Mtb*), a bacterial pathogen that causes dysregulated cell death and inflammatory signaling in the lung, leads to upregulation of MLKL protein expression, but this does not contribute to the amount of cell death, nor the overall inflammatory reaction associated with tuberculosis [42]. Necroptosis relies on inhibition of caspase-8. As a result, necroptotic death is more likely to be relevant during infections caused by pathogens that possess caspase-8 inhibitors, such as poxviruses [15, 60, 61]. In fact, to counteract necroptosis, numerous viruses have also evolved inhibitors of the necroptosis pathway to prevent cell lysis and sustain their replicative niche [60, 62, 63]. Nonetheless, SARS-CoV-2 is not known to encode a caspase-8 inhibitor, which further supports the notion that necroptosis does not play a prominent role in this context.

Although the lung has been shown to be a plausible site for necroptosis to occur, as recently observed by Lu et al. in the context of chronic obstructive pulmonary disease [64], our results suggest that necroptosis in the lungs cannot be induced by SARS-CoV-2 infection. The lack of phosphorylated MLKL and the fact that genetic deletion of MLKL did not alter disease parameters in our SARS-CoV-2 mouse models mimicking mild and severe COVID-19 indicates that, while necroptosis is primed upon SARS-CoV-2 infection, MLKL dependent membrane disruption, cytokine release and death do not contribute to disease. Targeting of MLKL, or other methods of interfering with necroptosis, are unlikely to represent therapeutic avenues for the management of COVID-19.

## Materials and methods

### SARS-CoV-2 strains

SARS-CoV-2 VIC2089 clinical isolate (hCoV-19/Australia/VIC2089/2020) was obtained from the Victorian Infectious Disease Reference Laboratory (VIDLR). Mouse-adapted (P21) strain was achieved by serial passage of VIC2089 through successive cohorts of young C5BL/6 mice. Briefly, mice were infected with SARS-CoV-2 clinical isolate intranasally. At 3 dpi, mice were euthanised and lungs harvested and homogenized in a Bullet Blender (Next Advance Inc) in 1 mL Dulbecco’s modified Eagle’s medium (DMEM) media (Gibco/ThermoFisher) containing steel homogenization beads (Next Advance Inc). Samples were clarified by centrifugation at 10,000 × *g* for 5 minutes before intranasal delivery of 30 µl lung homogenate into a new cohort of naïve C57BL/6J mice. This process was repeated a further 20 times to obtain the SARS-CoV-2 VIC2089 P21 isolate. Lung homogenate from all passages was stored at −80 °C. Work with SARS-CoV-2 clinical isolates was approved for use under Physical Containment Level 3 (PC3) conditions by the WEHI internal biosafety committee. All mouse strains and procedures were reviewed and approved by the WEHI Animal Ethics Committee (AEC) and Office of the Gene Technology Regulator (OGTR) under relevant institutional guidelines, the Prevention of Cruelty to Animals Act 1986 and associated regulations, and the Australian Code of Practice for the Care and Use of Animals for Scientific Purposes 2013.

### SARS-CoV-2 murine infection

Six- to eight-week-old or 6–8-month-old (aged) WT C57BL/6J or gene-targeted *Mlkl* [16] (on a C57BL/6J background) mice were anesthetized with methoxyflurane and inoculated intranasally with 30 μl SARS-CoV-2. Virus stocks were diluted in serum free DMEM to a final concentration of 10^4^ TCID50/mouse. After infection, animals were visually checked and weighed daily for a minimum of 10 days. Mice were euthanized at the indicated times post-infection by CO_2_ asphyxiation. For histological analysis, animals were euthanised by cervical dislocation. Lungs were collected and stored at −80 °C in serum-free DMEM until further processing. WT and *Mlkl*^−/−^ mice were from different colonies and co-housed in the same facility at least a week before commencement of experiments. Animals were age- and sex-matched within experiments (both sexes were used) and randomly allocated to infection groups. Investigators were not blinded to groups as they were involved in all aspects of group allocation, data collection and analysis. Experimental mice were housed in individually ventilated microisolator cages under level 3 biological containment conditions with a 12-h light/dark cycle, and provided standard rodent chow and sterile acidified water *ad libitum*. Animals that did not show productive infection (TCID50 under the limit of detection) were excluded in experiments that compared viral response (cytokine analysis and histology) between different mouse genotypes.

### Measurement of viral loads via 50% tissue culture infectious dose (TCID_50_)

TCID_50_ was performed as previously described in [65]. Briefly, African green monkey kidney epithelial Vero cells, purchased from ATCC (clone CCL-81), were seeded in DMEM + 10% FCS in flat bottom 96-well plates (1.75 × 10^4^ cells/well) and left to adhere overnight at 37 °C/5% CO_2_. Cells were washed twice with PBS and transferred to serum-free DMEM containing TPCK trypsin (0.5 µg/mL working concentration). Infected organs were defrosted, homogenized, clarified by centrifugation at 10,000 × *g* for 5 min at 4 °C and supernatant was added to the first row of cells at a ratio of 1:7, followed by 9 rounds of 1:7 serial dilutions in the other rows. Cells were incubated in serum-free DMEM containing TPCK trypsin at 37 °C/5% CO_2_ for 4 days until virus-induced cytopathic effect (CPE) was scored. TCID_50_ was calculated using the Spearman & Kärber algorithm as described in [65].

### Histological analysis and antigen staining

Organs were harvested and fixed in 4% paraformaldehyde (PFA) for 24 h, followed by 70% ethanol dehydration, paraffin embedding and sectioning. Slides were stained with either hematoxylin and eosin (H&E), or immunohistochemically with anti-CD3 (1:500, Agilent A045201), anti-MPO (1:1000, Agilent A039829), anti-F4/80 (1:1000, WEHI in-house antibody) or anti-SARS-CoV-2 nucleocapsid (1:4000, abcam ab271180) using the automated Omnis EnVision G2 template (Dako, Glostrup, Denmark). Dewaxing was performed with Clearify Clearing Agent (Dako) and antigen retrieval with EnVision FLEX TRS, High pH (Dako) at 97 °C for 30 min. Primary antibodies were diluted in EnVision Flex Antibody Diluent (Dako). and incubated at 32 °C for 60 min. HRP-labeled secondary antibodies were applied at 32 °C for 30 min. Slides were counter-stained with Mayer Hematoxylin, dehydrated, cleared, and mounted with MM24 Mounting Medium (Surgipath-Leica, Buffalo Grove, IL, USA). Slides were scanned with an Aperio ScanScope AT slide scanner (Leica Microsystems, Wetzlar, Germany). Histology images were scored by an American board-certified pathologist (Smitha Rose Georgy) using a histopathological scoring system (0–2) to grade histological changes based on H&E staining. The score was based on the average of the following parameters [66]: Inflammatory cells in the alveolar space (0 = none, 1 = 1–5 cells, 2 **≥** 5 cells), inflammatory cells in the interstitial space/septae (0 = none, 1 = 1–5 cells, 2 **≥** 5 cells), presence of hyaline membranes (0 = none, 1 = 1 membrane, 2 **≥** 1 membrane), proteinaceous debris in air space (0 = none, 1 = 1 instance, 2 **≥** 1 instance) and alveolar septal thickening (0 > 2x mock thickness, 1 = 2–4x mock thickness, 2 **≥** 4x mock thickness).

### Lung cytokine and chemokine analysis

Lungs were thawed, homogenized and clarified by centrifugation at 10,000 × *g* for 5 min at 4 °C. Supernatants were pre-treated for 20 min with 1% Triton-X-100 for viral deactivation and the Cytokine & Chemokine 26-Plex Mouse ProcartaPlex Panel 1 (EPX260-26088-901) was used as described in the manufacturer’s manual. 25 µL of clarified lung samples were diluted with 25 µL universal assay buffer, incubated with magnetic capture beads, washed, incubated with detection antibodies and SA-PE. Cytokines were recorded on a Luminex 200 Analyser (Luminex) and quantitated via comparison to a standard curve. For analysis of “cytokine storm”, 26 chemokines/cytokines from each animal were summed and compared between relevant groups.

### Western blot

Total cell protein was isolated from whole mouse lungs using cell lysis buffer. Absolute protein content of clarified lysates was determined by Bradford assay (Bio-Rad, Hercules, CA, USA), and equal quantities (20–50 μg) of total protein were separated under denaturing and reducing conditions (with 5% β-mercaptoethanol) using 4–12% SDS-PAGE gels (Life Technologies). Proteins were transferred onto nitrocellulose membranes, blocked with either 5% skim milk (Devondale, Brunswick, Australia) or 5% BSA (for phospho-proteins) in PBS with 0.05% Tween-20 (PBST) for 1 h, and detected using the following primary antibodies: rat anti-MLKL (3H1; available from Merck Millipore, Bedford, MA), rabbit anti-p-MLKL (phospho S345) (EPR9515; Abcam, Cambridge, UK), rabbit anti-phospho-T231/S232 mouse RIPK3 (Genentech; clone GEN135-35-9 [67]), rat anti-mouse RIPK3 (clone 8G7; produced in-house [68], available from Millipore as MABC1595; rabbit anti-phospho-S166 mouse RIPK1 (Cell Signaling Technology 31122), rabbit anti-mouse or human RIPK1 (Cell Signaling Technology; clone D94C12), anti-SARS-CoV-2 nucleocapsid (1:4000, abcam ab271180) and rabbit anti-GAPDH (Cell Signaling Technology, 14C10). HRP-conjugated goat secondary antibodies (Southern Biotech, Birmingham, AL, USA) were then applied to membranes, which were subsequently incubated with Amersham ECL Prime Western Blotting Detection Reagent (GE Healthcare) and imaged using a ChemiDoc Touch Imaging System (Bio-Rad). TSI control for mouse samples was a lysate derived from mouse bone marrow derived macrophages (BMDMs) treated for 6–9 h with 100 ng/mL mouse TNF (Biolegend), 10 μM SMAC mimetic (Birinapant; Tetralogic Pharmaceuticals) and 40 μM pan-caspase inhibitor QVD-OPh (Sigma-Aldrich, St. Louis, MO).

### Quantification and statistical analysis

Statistical analyses were performed using Prism v9.3.1 software (GraphPad Software, Inc.). Unpaired two-tailed t-tests were used for normally distributed data for comparisons between two independent groups. Data that violated the assumption of normality were transformed by generating log_10_ prior to statistical analysis. Bars in figures represent the mean (±SD) and each symbol represents one mouse. Sample sizes (n), replicate numbers and significance can be found in the figures and figure legends. For all statistical significance indications: *p < 0.05; **p < 0.01; ***p < 0.001; ****p < 0.0001; ns not statistically significant (*p* > 0.05). Animal studies were performed in accordance with the 3Rs (Replacement, Reduction and Refinement), the smallest sample size was chosen that could give a significant difference (less than 0.05 type 1 error probability at 0.8 power).

Statistical analysis of cytokine data consisted of Wilcoxon rank sum test between group medians, with Bonferroni adjustment for multiple comparisons. Boxplots in figures depict the median and interquartile ranges.

### Reporting summary

Further information on research design is available in the Nature Research Reporting Summary linked to this article.

### Supplementary information

Supplemental Figure Legends

Supplemental Figure 1

Supplemental Figure 2

Supplemental Figure 3

Original Data File

Reporting Summary

## Data Availability

Data obtained during this study are included in this published article. Any additional information required to reanalyze the data reported in this paper is available from the lead contact upon request. Original high resolution histological images reported in this paper will be shared by the lead contact upon request. Uncropped western blots are available in the supplementary materials.
