# Supplementary material for: Necroptosis does not drive disease pathogenesis in a mouse infective model of SARS-CoV-2 in vivo
Source: Cell Death Dis. 2024 Jan 30;15(1):100. doi: 10.1038/s41419-024-06471-6 (PMC10825138; doi:10.1038/s41419-024-06471-6)
Supplement: Supplementary file 1 — Supplemental Figure Legends [file 41419_2024_6471_MOESM1_ESM.docx]

# Supplementary Figure Legends

**Figure S1. *Mlkl* knockout does not affect immune cell infiltration in the lungs of mice infected with a clinical SARS-CoV-2 isolate. (A)** Representative images of immunohistochemistry (IHC) stained lungs with CD3 (T cells), F4/80 (macrophages) and myeloperoxidase (MPO, neutrophils). Mice were challenged intranasally with 10^4^ TCID50 of P2 SARS-CoV-2 and lungs were collected and fixed for histological analysis at 2 and 4 dpi. Images are representative of at least 3 mice. Scale bars = 500 µm.

**Figure S2. Severe disease caused by P21 is dependent on mACE2, but unrelated to necroptotic death. (A-B)** Lung viral loads of wild-type (WT), and *Ace2^-/^*^-^ mice 3 days post-infection with 10^4^ TCID50 of **(A)** P2 or **(B)** P21 (n=4, unpaired two-tailed student’s t-test after log10 transformation). **(C)** Mice were challenged intranasally with 10^4^ TCID50 of P21 SARS-CoV-2 and animals were weighed daily for 10 days post-infection (dpi) (n=7-8 mice per group) **(D)** Cytokines and chemokines in lung homogenates of 6–8 week-old C57BL/6 or *Mlkl*^-/-^ mice 3 days after challenge with P21 or mock (n=4 mice per group). Wilcoxon rank-sum tests, with Bonferroni adjustment for multiple comparisons, were performed between groups. ****p < 0.0001.

**Figure S3. Lack of necroptosis does not impair immune cell infiltrates during severe disease, or protective immunity to re-challenge with a clinical SARS-CoV-2 isolate. (A)** Lungs of P21 infected animals were collected and fixed for histological analysis at 4 dpi. Representative images of hematoxylin and eosin (H&E) and immunohistochemistry (IHC) with SARS-CoV-2 nucleocapsid, CD3 (T cells), F4/80 (macrophages) and myeloperoxidase (MPO, neutrophils) are shown. Histological images are representative of at least 3 animals. Scale bar = 500 µm. **(B-C)** H&E staining of P21 infected WT and *Mlkl^-/-^* lungs animals at 3 dpi were analysed by a pathologist at scored based on **(B)** signs of disease severity and **(C)** percentage of lung lobes affected by interstitial pneumonia **(D)** Mock or P2-infected C57BL/6 and *Mlkl*^--/-^ mice were rechallenged 28 days later with P2 and analysed 3 days post re-challenge for lung viral load (TCID50) (n=9-10 mice). One-way ANOVA with multiple comparisons after log_10_ transformation was performed on primary data. Mean ± SD are shown. ****p < 0.0001.
