# Supplementary figures and images for: Necroptosis does not drive disease pathogenesis in a mouse infective model of SARS-CoV-2 in vivo
Source: Cell Death Dis. 2024 Jan 30;15(1):100. doi: 10.1038/s41419-024-06471-6 (PMC10825138; doi:10.1038/s41419-024-06471-6)

Figure S1

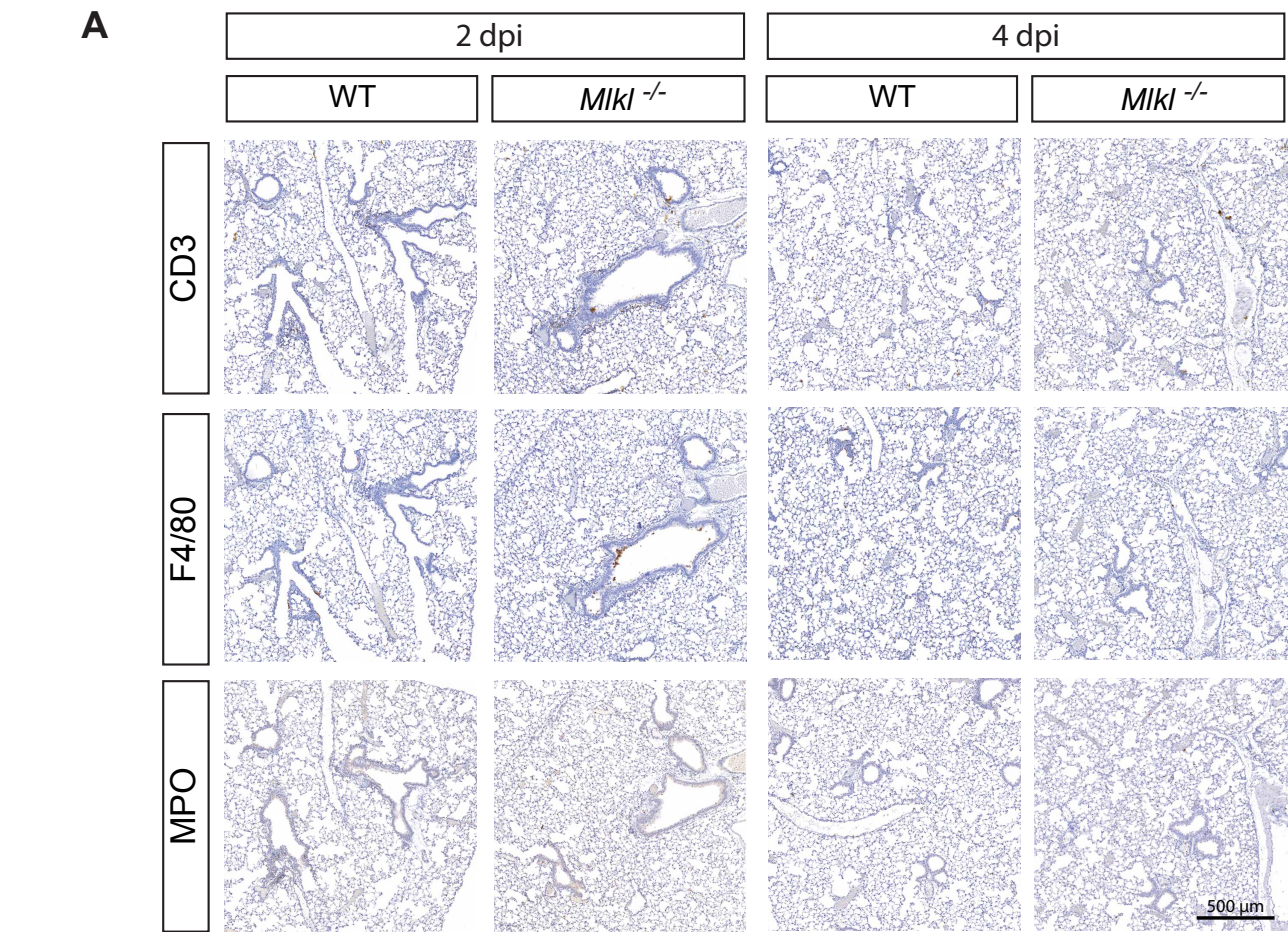

Supplement: Supplementary file 2 — Supplemental Figure 1 [file 41419_2024_6471_MOESM2_ESM.pdf]

Figure S2

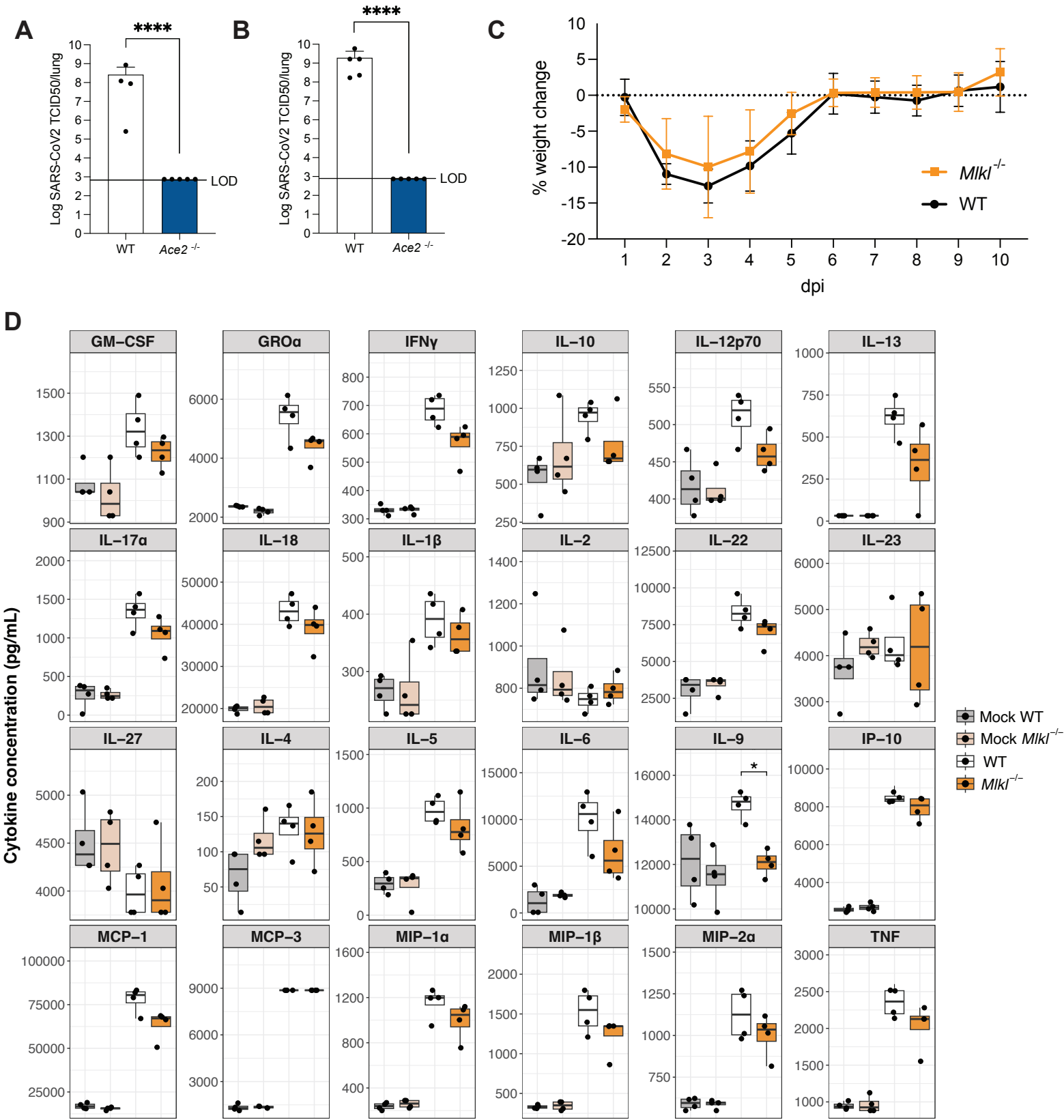

Supplement: Supplementary file 3 — Supplemental Figure 2 [file 41419_2024_6471_MOESM3_ESM.pdf]

Figure S3

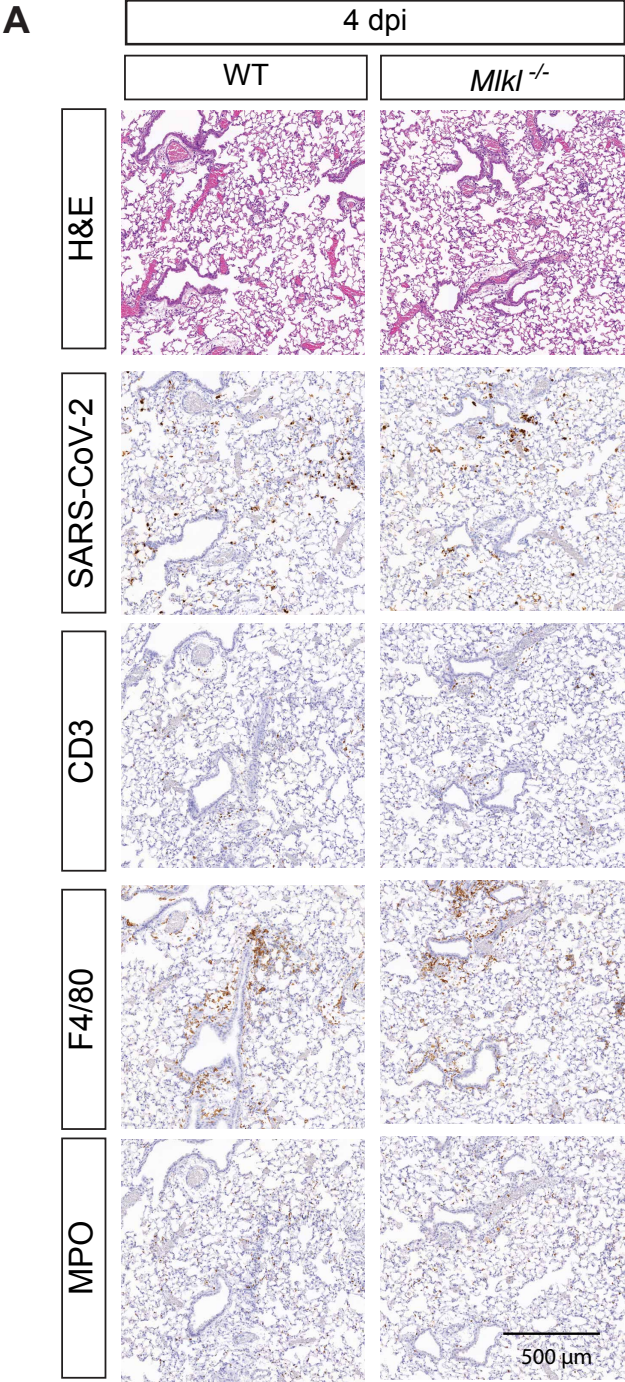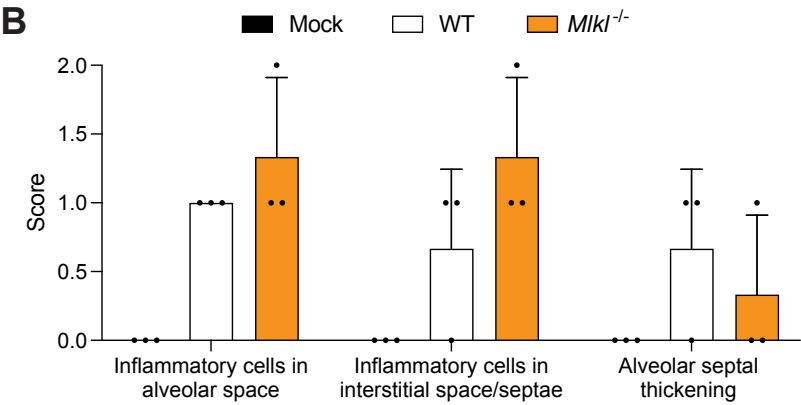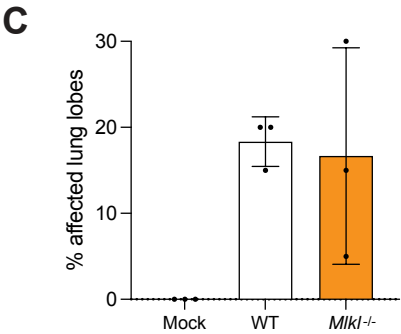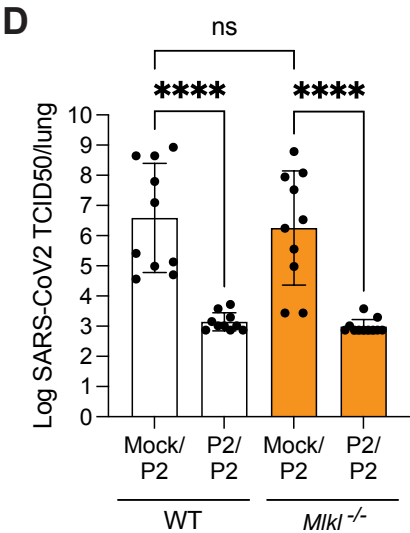

Supplement: Supplementary file 4 — Supplemental Figure 3 [file 41419_2024_6471_MOESM4_ESM.pdf]
