# Supplementary material for: Necroptosis does not drive disease pathogenesis in a mouse infective model of SARS-CoV-2 in vivo
Source: Cell Death Dis. 2024 Jan 30;15(1):100. doi: 10.1038/s41419-024-06471-6 (PMC10825138; doi:10.1038/s41419-024-06471-6)

Original Data Files

Fig. 1D

p-MLKL

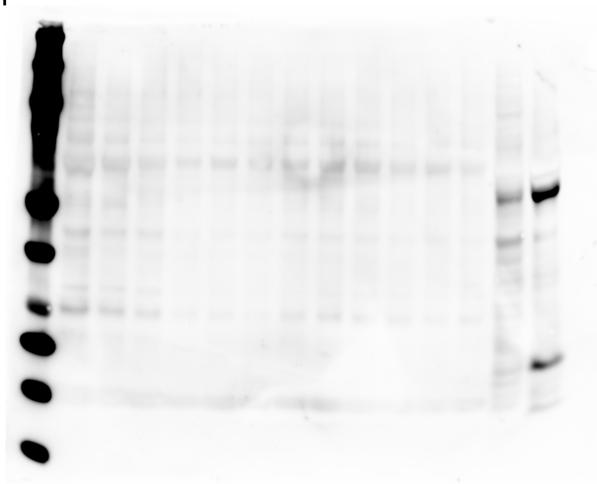

↓ stripping

MLKL and GAPDH

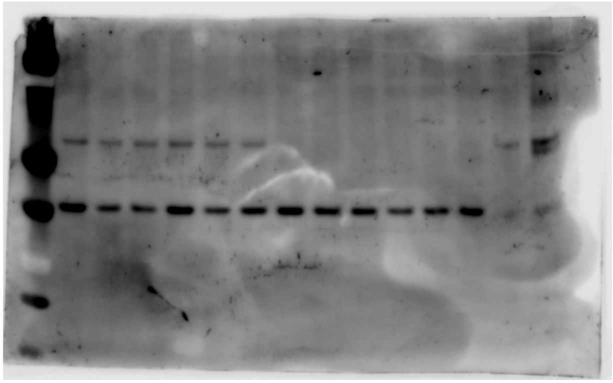

p-RIPK1

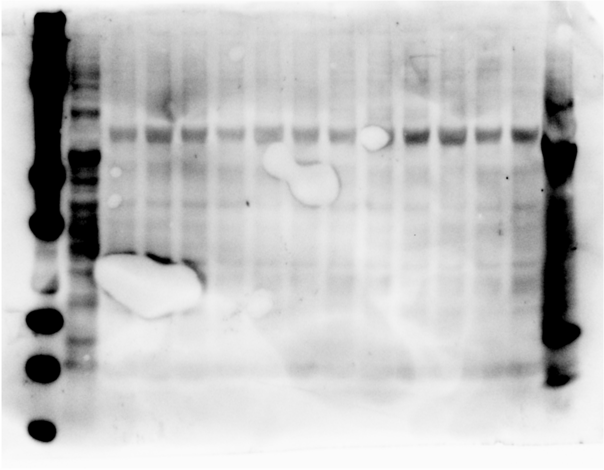

p-RIPK3

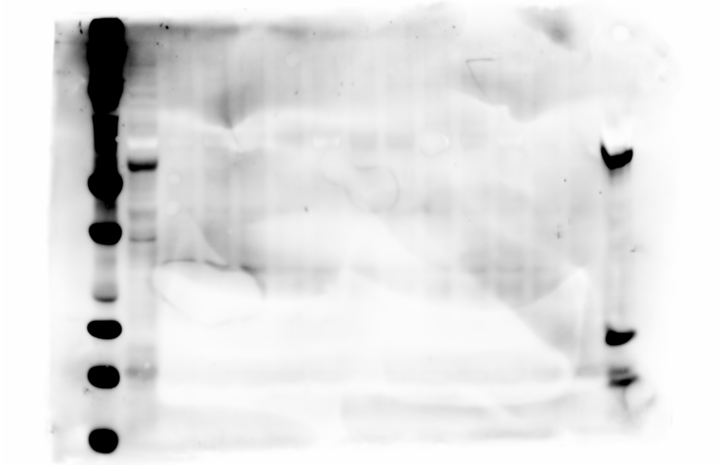

GAPDH (p-RIPK1 and p-RIPK3)

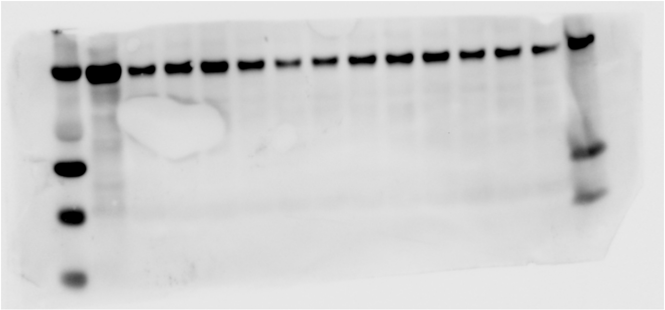

RIPK3

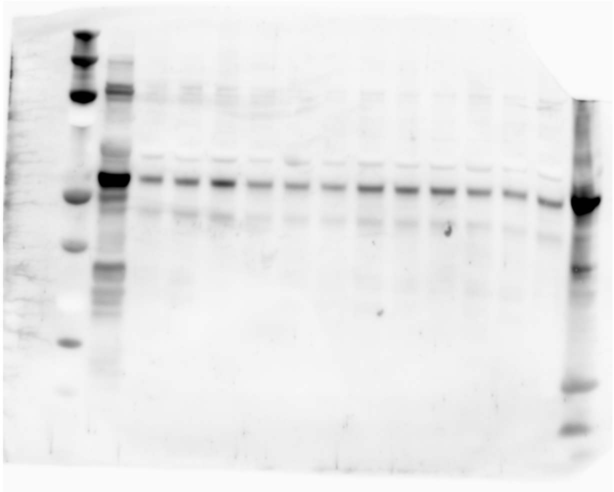

stripping

RIPK1

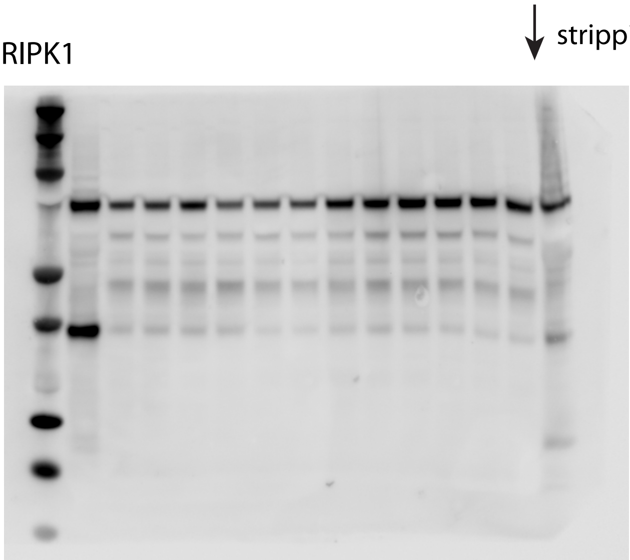

GAPDH (RIPK1 and RIPK3)

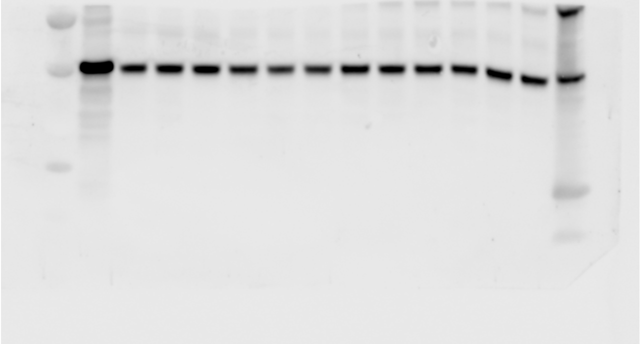

Fig. 2D

SARS-CoV-2

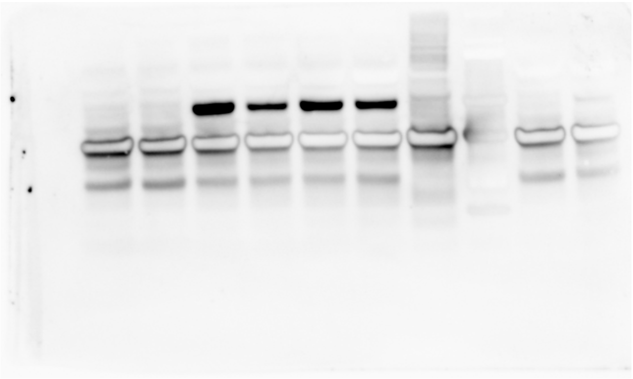

p-MLKL

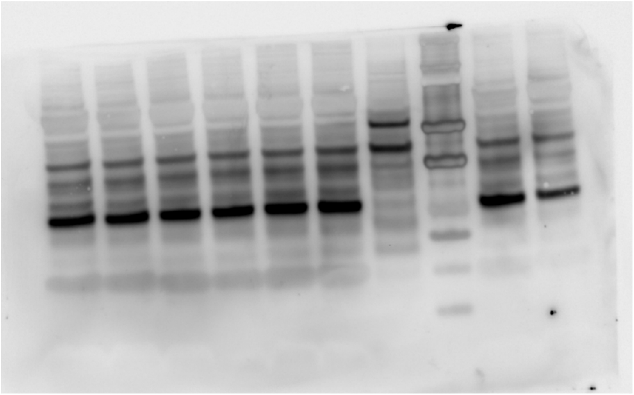

GAPDH (p-MLKL)

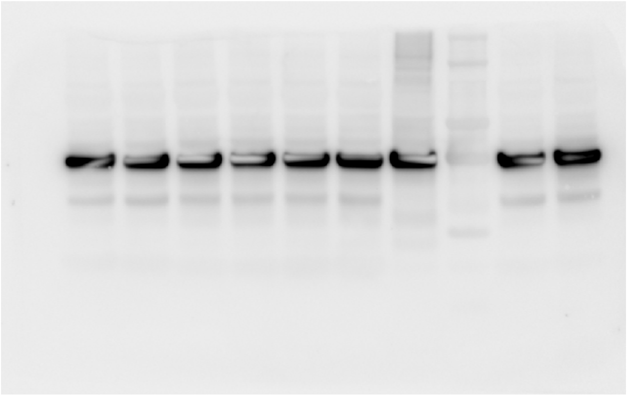

MLKL

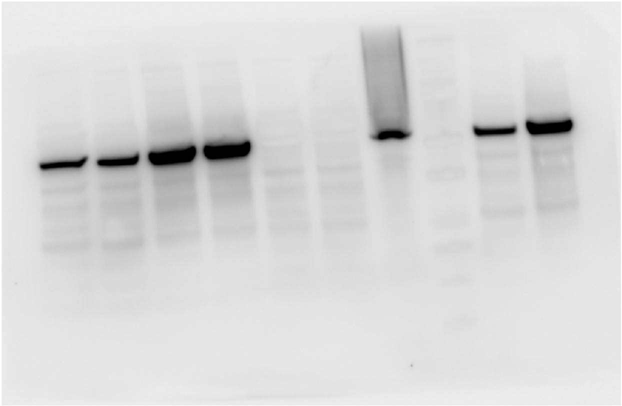

MLKL and GAPDH

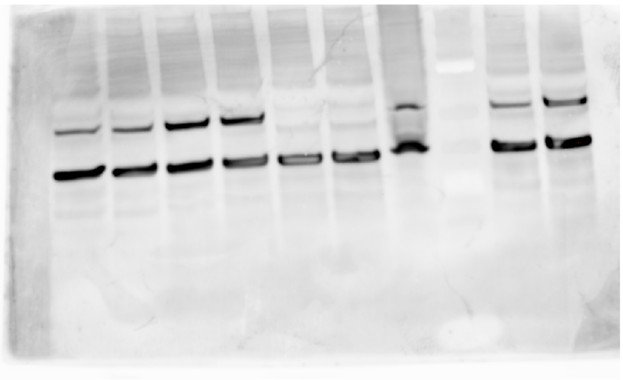

p-RIPK1

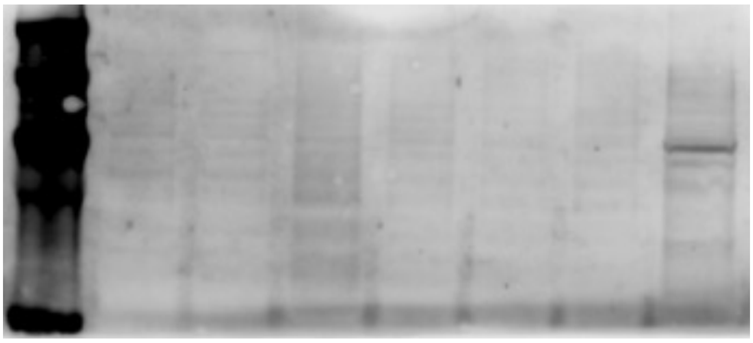

RIPK1

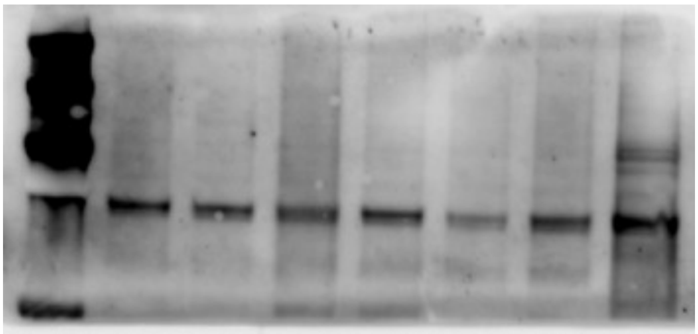

GAPDH (p-RIPK1, RIPK1)

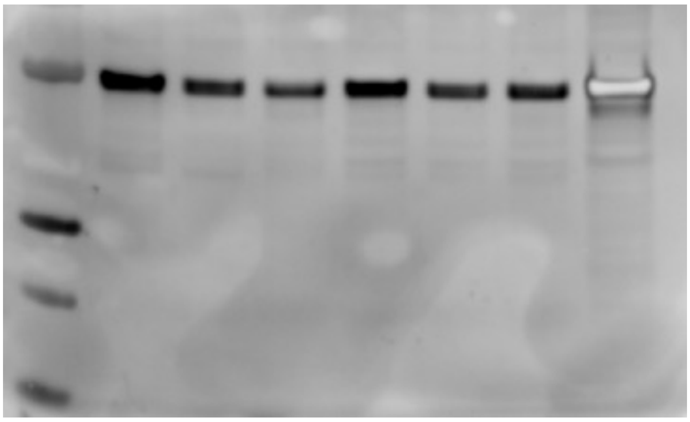

p-RIPK3

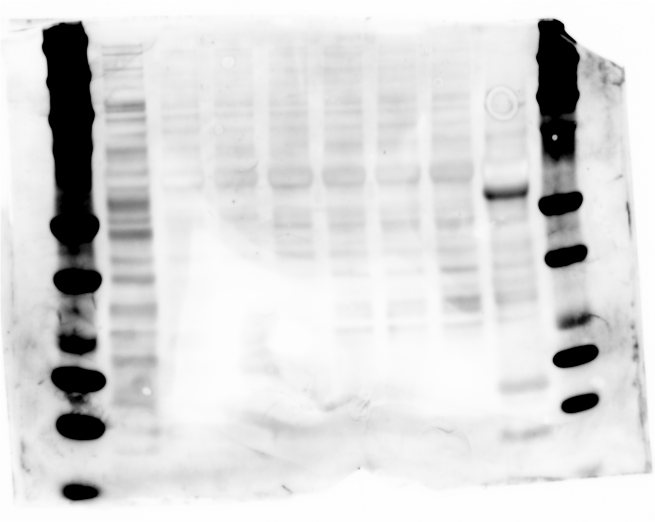

GAPDH (p-RIPK3)

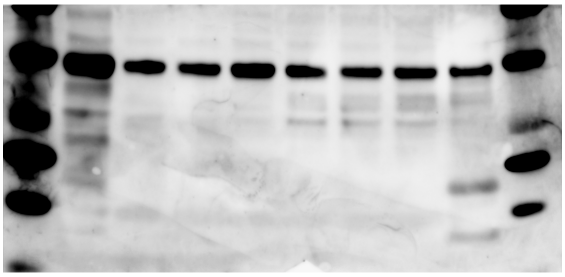

RIPK3

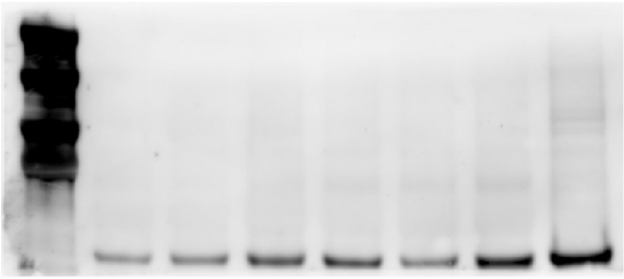

GAPDH (RIPK3)

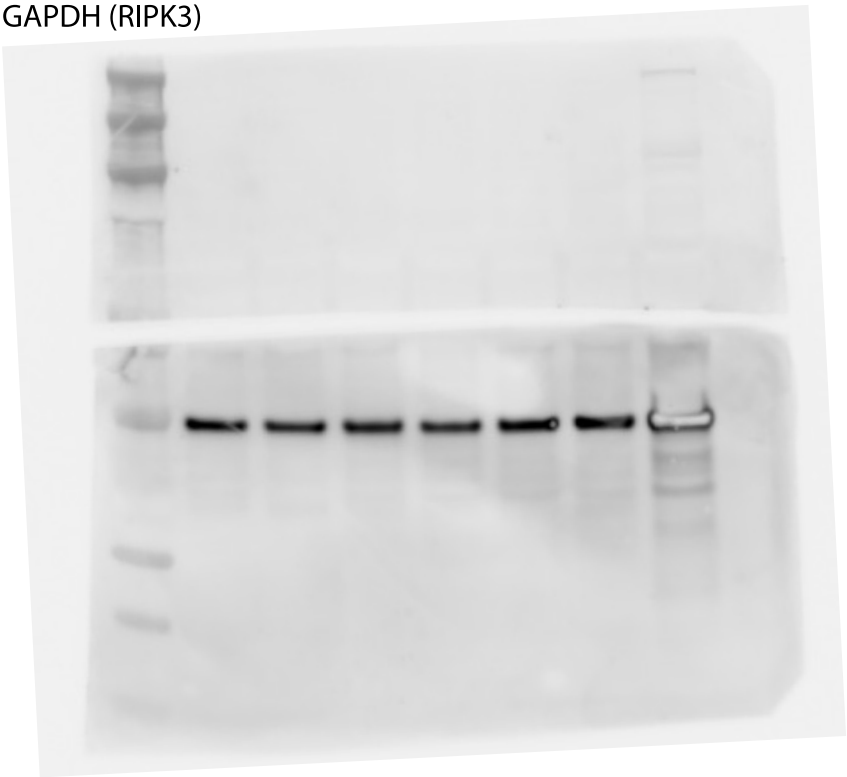

Supplement: Supplementary file 5 — Original Data File [file 41419_2024_6471_MOESM5_ESM.pdf]
